# Fevers from the East

**Published:** 1919-12-13

**Authors:** 


### FEVERS FROM THE EAST.

### A Plea for Greater Care and Accuracy.

In recent hospital practice in this country the physician cannot have failed to notice that there are quite a number of pyrexial cases finding their way into the medical wards, the exact origin and diagnosis of which is by no means easy to decide if the attempt be made along the lines commonly accepted in pre-war times. Undoubtedly many of these are remotely or closely associated with the patient's war-time travels. And even when acting in full realisation of this fact it is still difficult for any but the most experienced, satisfactorily to sort out the many curious forms in which these relapses or reminders present themselves. Both expert clinicians and the workers in the laboratory are faced with many perplexing problems and this applies more strongly to the less advantageously placed general practitioner. In spite of the latest Governmental activity in bringing in adequate notification measures and in attempting more widely to disseminate knowledge on tropical disease, the untravelled medical man in this country is for the moment working under no little handicap in this respect, and one feels that it is perhaps worth while drawing attention to a few well-known but not always appreciated facts in connection with fevers from the East.

### General Considerations.

In an official memorandum published at the close of hostilities it was well stated that there is a time-honoured warning to the effect that when you see a sick child you should not always think of a grey powder. In the same way, when a case of pyrexia from the tropics is encountered do not always think of malaria. The medical man is only too apt after the campaigns in malarious countries to consider every such fever as malarial, to the exclusion of all other forms of pyrexia. True it is that in such regions this disease is the most common cause of fever; that with great frequency it complicates other disorders, and moreover, that it is the most protean of all fevers and may simulate well-nigh every febrile complaint. Hence we have the obvious necessity in all such cases, wherever and whenever possible, of examining directly the patient's blood film, but we must go further.

This is but one example, and in so short a note it is impossible to touch on more than the outskirts of so huge a subject as tropical and subtropical pyrexias; therefore, all that is here attempted is to give the practitioner some idea of what he is likely to encounter, and particularly to indicate how such cases of fevers should be approached for diagnosis. It must be remembered that in the country of origin these fevers may be either infective or non-infective, and that although most of the war-zone pyrexias belong to the former class, yet the latter do arise from the effects of sun, chill, and fatigue, and though simple at the outset may meanwhile have lowered the resistance of the body, paving the way for the infective entry which is thus readily masked, and therefore passed over by the original medical observer. Here at home we can largely omit the non-infective type and then view our unknown fevers as probably due to the invasion or presence of a living parasite, and unless this be ultramicroscopc, as in dengue, or though possibly visible, as yet unidentified as in trench fever, it should be possible, somehow, to find it.

### A Straightforward Principle.

To begin simply, let us remember that the parasite is either bacterial, protozoal, or helminthic, and that the infection may be local or general, with a subdivision of internal and external for the former. An external local lesion may be simply attacking the skin and producing fever, while an internal localisation may be seen in dysentery or in hepatic abcess. In generalisation there may be a septicæmia, as in typhoid or plague; a specific blood condition, as with malaria; or a disease affecting cellular elements, both in the blood and internal organs, as in kala-azar. Speaking broadly, local reaction follows local infection, and this reveals itself by symptoms and physical signs, and a search of the local secretion or discharges should lead one directly to the parasite. Again, let us appreciate the evidence obtained from blood-cell changes owing to an absorbed toxin such as the eosinophilia in worm infections.

The best working plan is, therefore, to exclude the local infection, and to do this a more careful, thorough physical examination is necessary than is often granted. Some guidance at least will generally be found to the spot at fault, and a laboratory diagnosis may be at once possible. But these home cases of ours are rarely so simple, and the summary of advice as published by the R.A.M.C. is so appropriate that we may quote freely: "Direct evidence may not be obtainable, and in certain instances it is necessary to carry out blood tests to make sure that the organism isolated is the cause of the condition. There may be two conditions, one masking the other. Physical signs may not have developed at the time of examination, and hence the latter must be repeated. If, however, the case is properly gone into, and the services of a competent bacteriologist fully utilised, it is more than likely that the local condition producing the fever will be detected and diagnosed. It might be imagined that in any general infection of the circulation it would be easy to find the parasite causing the pyrexia, but this is not always the case. We know, for example, that the *bacillus typhosus* may leave the blood stream after the first few days of the fever, and that at certain periods the parasite of malignant malaria is unlikely to be found in the peripheral blood. Micro-organisms tend to lodge in certain backwaters where it may not be easy to get at them, and hence the evidence of their presence may be indirect (agglutination tests), or they may only be discovered in excretions (*B. dysenteriæ* in stools).

**Fevers from the East**—(*continued*).

In general infections the physical examination of the patient is again very important, but it must go hand in hand with the laboratory investigation, and the latter must be thorough. It suffices here to say that it should include an ordinary blood examination for parasites (repeated, if necessary, on several occasions) by the thick film method, as for malaria and relapsing fever, or by culture, as for enteric. The red and white cells should be counted, and the exceedingly useful differential leucocyte count should be carried out, at least 500 cells being enumerated. A spleen infection may be diagnosed by splenic puncture, and hepatic abcess by liver puncture, while the blood condition may indicate changes in the bone marrow. The stools and urine must *always* be examined, especially the former. If the blood is the life, the stool is the expression of life, at least in one sense. The examination of the sputum should not be neglected, even when tubercle is not suspected.

THE LESSON DERIVED.

And so the plea becomes to all intents that for the clinician the important thing is to remember that in tropical work the laboratory, or at least the microscope, is an absolute necessity. The greatest consultant may fail utterly without its aid, and in similar circumstances it is not too much to say that the diagnosis of the tyro is mere guesswork. Such statements may strike the reader as being the veriest platitudes, but they may rest assured that it was not only in the earliest phases of the war in the East that great and frequent difficulty was experienced in persuading medical officers to realise this truth.

To apply the lesson to our work at home two distinct sets of circumstances must be considered, and from first-hand experience we venture to set down one of the most outstanding causes of failure to attain the ideal. In the first place, there is the big institution with its perfected laboratory, staff and equipment and its big clinical "firms." In practice the very magnitude of its organisation often detracts from its efficiency when dealing with these cases. The routine specimen, or serus, is so often accompanied by such utterly inadequate descriptions of clinical details and observations required that any laboratory investigation is conducted in anything but an enlightened atmosphere. Not that the physician in charge has failed to give the fullest verbal directions to his "firm," but rather that the fatal habit is developed in the press of teaching requirements of leaving the simple, yet vital, duty of "filling in the form" to a junior student or the sister. We hear on every side accounts of increased linking-up of departments in our great hospitals, but one would strongly suggest that the links must be of more refined material than the average ward clerk. Under present conditions a host of discrepancies appear, and except with those cases where the physician learns all and every detail of the laboratory findings and the pathologist learns beforehand the precise clinical situation, there is no doubt that but half of the possible efficiency is attained. In practice one fears that our obscure pyrexial cases suffer not unfrequently from this circumstantially caused neglect. Similarly in private practice, with the practitioner's specimens sent from a distance to a central laboratory. Full postal interchange of medical and scientific details can be carried out, but how rarely is it correctly done? The writer knows something of the workings of both trade and institutional laboratory departments, and can only affirm that the greatest failures, unbelievably great, and disastrous failures for the patient's ultimate welfare, occur simply because material is sent in, perhaps ideally collected, packed, etc., but accompanied by the merest outline of what is required—and by less as to what is expected.

THE FUTURE.

A little belatedly perhaps has developed centralised laboratory and clinical control of Ministry of Pensions cases falling in the tropical diseases category, and already it is obvious that not only this centralisation but the typical transference by unending forms of the minutest details concerning every patient is of far greater value than many lay critics would have us believe. A multiplicity of forms, interminable queries and spaces which must be filled, and the whole procedure, characteristic of Government action, has been so widely and so scathingly denounced by thousands who took either an active or inactive part in the war, that it is just worth while here to draw attention to an instance, small though it is, where precision, almost laboured in its character, is the only policy which can ever be pursued with success. Let us realise that minute accuracy is every bit as necessary in scientific as in administrative organisation.

